# The Pathological Anatomy of Erysipelas

**Published:** 1875-12

**Authors:** L. Putzel

**Affiliations:** New York


### THE PATHOLOGICAL ANATOMY OF ERYSIPELAS.

BY L. PUTZEL, M. D., NEW YORK.

During the last few years medical literature has been teeming with discussions on the bacterial origin of disease. Of late the relation of these organisms to the production and propagation of erysipelas have been an especial subject of investigation, and, during the autumn of last year, Von Recklinghausen and his pupil, Lukomsky, affirmed, as the result of their labors in this field, that micrococci were the active agents in the development of erysipelas. In view of the statements recently made in opposition to these views by such distinguished pathologists as Robin and Billroth, the following *resumé* of the elaborate researches of Dr. Ponfick, assistant in the surgical clinic at Heidelberg, which were made in 1866, but which have never before appeared in English, will probably prove of interest to our readers. It is condensed from a series of articles in the *Deutche Klinik:*

Dr. Ponfick examined eleven cases of erysipelas, of which only three were typical examples of the disease, five being attended with serious complications, while in three cases the erysipelatous inflammation played an unimportant part in producing the fatal termination. In addition, Dr. Ponfick inoculated the sero-purulent liquid from an erysipelatous vesicle under the skin of two rabbits, and after a short illness, attended with high temperature, post-mortem examination showed appearances identical with those now to be described as having been found in the fatal cases of erysipelas.

The condition of the organs in the latter disease was as follows:

*Heart.*—Flabby, friable, pale color. The transverse striæ of the muscular fibre had partly disappeared. There was finely-granular, and, in marked cases, fatty degeneration of the fibrillæ.

*Muscles* of the trunk and extremities were in the same condition as the heart, but the morbid changes were not so marked in degree.

*Spleen.*—Some degree of enlargement and softening was always present. The parechyma was bright red, soft, often almost fluid; Malpighian bodies mostly plainly visible and hyperplastic.

*Partal and Mesenteric Glands.*—Usually swollen, injected, and hyperplastic.

*Liver.*—This presented the evidences of acute parenchymatous inflammation (cloudiness, enlargement, paleness, friability). The liver-cells were swollen, and contained albuminous and fatty granules. The nuclei were frequently indistinct, sometimes absent. In marked cases the cells were completely filled with fat and pigment-granules.

*Kidneys.*—Had likewise undergone acute parenchymatous inflammation (cloudliness, enlargement, pallor, friability); they were usually anæmic, especially the cortex. Epithelium of tubules in first stages somewhat swollen, dull, shining, and cloudy from the presence of albuminous and fatty particles. In advanced stages the cells were filled with a greater number of fatty particles; the nuclei were mostly pale and cloudy, sometimes increased in number, sometimes absent.

*Blood-Vessels.*—The endothelial cells were swollen and cloudy; nucleus pale and indistinct, sometimes two present, sometimes none. In more advanced cases the cells contained well-marked, sharply-refracting oil-granules, and were sometimes entirely degenerated. The tissue of the tunica intima was infiltrated with large and small fat-granules, chiefly diffused between the bundles of elastic fibres; a considerable amount of free fat was also present. In only two cases was the middle coat examined, and in these muscular fibres were clouded by numerous fine granules.

Changes similar to those just described were also found in the organs of those who died from diseases other than erysipelas, but which were attended by prolonged high fever.

It is, therefore, highly probable that the blood-vessels (three inner coats), heart, muscles, and large glandular viscera, become affected by acute parenchymatous inflammation in erysipelas, as well as in other diseases accompanied by continued high temperature.

May not the hitherto inexplicable hemorrhages in the various organs and tissues of the body after severe and prolonged attacks of erysipelas, typhoid fever, variola, scarlatina, pyæmia, etc., stand in casual relations to the above described changes? Prof. Weber has shown that by the absorption of pyogenic matter the blood becomes on "inflammation-producing" fluid. It is, therefore, but natural that in erysipelas this quality of the blood should first act upon the vessels, and then upon the spleen, liver and kidneys—organs which are so necessary to the production of blood—*N. Y. Med. Jour.*

